# Clinical and radiological evaluation of maxillofacial and otorhinolaryngological manifestations of Hansen’s disease

**DOI:** 10.1038/s41598-022-19072-0

**Published:** 2022-09-01

**Authors:** Rachel Bertolani do Espírito Santo, Rachel Azevedo Serafim, Rafael Maffei Loureiro, Dâmaris Versiani Caldeira Gonçalves, Daniel Vaccaro Sumi, Ricardo Andrade Fernandes de Mello, Simon M. Collin, Patrícia D. Deps

**Affiliations:** 1 Postgraduate Programme in Infectious Diseases, Universidade Federal do Espírito Santo, Vitoria, Espírito Santo Brazil; 2 Imaging Department, Hospital Israelita Albert Einstein, São Paulo, Brazil; 3 Department of Internal Medicine, Federal University of Espírito Santo, Vitoria, Espírito Santo Brazil; 4 Department of Social Medicine, Centro de Ciências da Saúde, Universidade Federal do Espírito Santo, Avenida Marechal Campos, 1468. Maruípe, Vitoria, Espírito Santo CEP: 29047-105 Brazil

**Keywords:** Infectious diseases, Skin diseases

## Abstract

To characterize maxillofacial, otorhinolaryngological and oral manifestations of Hansen’s disease (HD), we conducted a cross-sectional study in 21 current patients attending the Unidade Básica de Saúde de Jardim América, Espírito Santo, Brazil and 16 former patients resident at Pedro Fontes Hospital using data from computed tomography imaging, rhinoscopy, and oroscopy. Maxillofacial characteristics were compared with 37 controls. Differences in bone alterations across the three groups were determined mainly by severe resorption/atrophy being more frequent in former HD patients, with severe resorption/atrophy of the anterior alveolar process of maxilla in 50.0% (8/16) of former patients, 28.6% (6/21) of current patients and 10.8% (4/37) of controls and of nasal bones and aperture in 31.3% (5/16) of former patients compared with 0/21 current patients and two controls. There were no substantial differences in otorhinolaryngological and oroscopic findings between the two patient groups. HD patients had more tooth loss than the age-matched control group. Maxillofacial, otorhinolaryngological and oroscopic finding scores were strongly correlated only in current HD patients. Correlation between otorhinolaryngological and maxillofacial scores suggests that protocols for HD patient assessment and follow-up could include otorhinolaryngological evaluation, with radiological imaging where necessary, subject to replication of our findings in a larger study.

## Introduction

Hansen’s disease (HD), also called leprosy, is a chronic mycobacterial infection that was a major scourge of Europe in the Middle Ages and is nowadays considered to be a ‘neglected tropical disease’, affecting poorer people in low- and middle-income countries in tropical and subtropical regions^1^. Hansen’s disease affects peripheral nerves and the skin, starting as a localized disease which, in susceptible individuals, can become generalized^2^. Involvement of the peripheral nerves causes motor and sensory dysfunction leading to disabilities especially in the hands, feet and eyes^3^. More than 200,000 new HD cases were diagnosed annually in the five years up to 2019, most of which (80%) occurred in India, Indonesia and Brazil, and which included 15,000 cases in children and 11,000 with disability^4^.

HD can involve bones of the hands, feet and face, leading to long-term disability and impacting adversely on quality-of-life^5–8^. *Mycobacterium leprae* infection of the nasal cavity can lead to collapse of the bridge of the nose, resorption of the central part of the maxilla and perforation of the hard palate^9,10^. Maxillofacial bone alterations, particularly in the severe form characterized as rhinomaxillary syndrome (RMS), cause secondary health problems and visible facial profile changes that contribute to the stigmatisation of persons affected by HD^11,12^.

We previously characterized maxillofacial bone alterations using computed tomography (CT) imaging with 3-dimensional (3D) reconstructions in a group of older (age 60–89 years) former HD patients in Brazil, finding that 4/16 had RMS and another 4 had partial RMS^13^. We suggested that clinical protocols for HD patient assessment and follow-up be extended to include otorhinolaryngological evaluation, supported by radiological imaging, primarily to prevent secondary complications.

The aim of the present study was to use these two methods—otorhinolaryngological evaluation, comprising rhinoscopy, nasal endoscopy and oroscopy, and radiological imaging, comprising CT scans of facial bones—to investigate maxillofacial bone manifestations of HD in the same group of former HD patients compared with current HD patients and non-HD controls, and otorhinolaryngological manifestations of HD comparing current with former HD patients.

## Methods

### Participants

Former HD patients were older persons (age 60+ years) resident at the Hospital Pedro Fontes (HPF), Cariacica, Espírito Santo, Brazil, who were invited to participate in this study when they required a medical assessment as part of routine health care during the period 09/2015-12/2016. Demographic data and medical history of Hansen’s disease were obtained by interviewing participants and from medical records held at Pedro Fontes hospital. Current HD patients were receiving specialist medical care (treatment or active follow-up) for HD at the Unidade Básica de Saúde de Jardim América (UBSJA), Cariacica, Espírito Santo, Brazil, during the period 09/2015 to 12/2016. A control group of non-HD patients was selected from among patients who underwent tomography during September 2020 in the radiology service of the Hospital Universitário Cassiano Antônio Moraes (HUCAM), Vitória, Espírito Santo, Brazil.

### Computed tomography (CT) imaging

All participants attended the HUCAM Radiology and CT Service for CT scans of facial bones. Images were acquired in a 64-section multidetector CT scanner (Aquilion, Toshiba Medical Systems Corp., Tochigi, Japan) with parameters: 120 kVp; 100 mAs; 1.125 mm spiral pitch factor. The field of view was limited to the maxillofacial area. CT images were examined independently by two radiologists with experience in head and neck imaging to identify the following maxillofacial structures abnormalities: osteitis of the orbital (frontal, zygomatic and lacrimal) bones; resorption of the nasal bones and anterior nasal spine; loss of sharpness of the pyriform aperture; perforation of the nasal septum; atrophy of the inferior and middle nasal turbinates; thinning with discontinuities of the hard palate; resorption of the alveolar process of the maxilla (anterolateral and posterior regions). Abnormalities, if detected, were graded as ‘severe’ or ‘mild-to-moderate’ and were recorded independently and in parallel by three researchers on assessment forms. Discordant opinions between the radiologists were resolved by consensus review. A maxillofacial bone alteration score was calculated as a sum of the score for each feature, where no change was scored 0, mild-to-moderate alterations 1, and severe 2. This yielded a theoretical range of 0–18.

### Rhinomaxillary syndrome (RMS)

Rhinomaxillary syndrome was diagnosed as previously described^13^. In brief, RMS is defined by changes to: (I) anterior nasal spine; (II) alveolar processes of maxilla; (III) nasal surface of the palatine process of the maxilla; (IV) oral surface of the palatine process of the maxilla; (V) nasal turbinates and septum; (VI) pyriform aperture; (VII) posterior alveolar margins of the maxilla^14^. Full or partial radiological RMS (rRMS) was based on assessment of the number and severity of alterations. ‘Fully met’ required severe resorption of anterior nasal spine (criterion I) and severe loss of sharpness of pyriform aperture and/or resorption of nasal bones (criterion VI) plus at least one of the other criteria rated severe and one rated mild-to moderate. Partial rRMS typically required 2–3 severe plus 2–3 mild-to-moderate bone alterations.

### Otorhinolaryngological examinations

Otorhinolaryngological examinations were performed by an otorhinolaryngologist (RS). Rhinoscopy and oroscopy were performed using halogen lamps and nasal speculum or wooden spatula. For patients with turbinate hypertrophy or septum deviation, in whom assessment was difficult, cotton with a vasoconstrictor solution (0.5% oxymetazoline) was placed in the nasal cavity for two minutes and the was rhinoscopy repeated. Patients in whom it was not possible to visualize the middle meatus in anterior rhinoscopy were evaluated through nasal endoscopy, performed with a flexible endoscope (Pentax 3.5 mm) without topical anaesthesia, after applying vasoconstrictor solution as described above. Otorhinolaryngological and oroscopic examination scores were calculated as a sum of the score for each feature, where normal/none was scored 0 and any other finding as 1. This yielded theoretical ranges of 0–13 for the otorhinolaryngological and 0–10 for the oroscopic scores.

### Data analysis

Bone alterations and clinical findings were summarised as frequencies and percentages or median and interquartile range (IQR). Differences between groups were tested using Fisher’s exact test for categorical variables and Kruskal–Wallis test for continuous variables (α = 0.05 for both tests). Correlations between maxillofacial bone alteration and clinical findings scores were estimated using Pearson’s correlation coefficients. Stata was used for all analyses (StataCorp. 2017. Stata Statistical Software: Release 15. College Station, TX: StataCorp LLC).

### Ethics approval

This study was approved by the Research Ethics Committee of the Health Sciences Center of the Federal University of Espírito Santo (nos. 1.101.787 issued 10/06/2015 and 4.248.419 issued 31/08/2020). Informed consent was obtained from all participants and the research was performed in accordance with national guidelines/regulations and in accordance with the Declaration of Helsinki.

## Results

### Participants

#### Former HD patients

Of the 16 former HD patients, 10 were female and 6 male, with median age 70 (range 60–89) years, age at diagnosis 20 (6–43) years, and time since diagnosis 46 (26–70) years. Original HD diagnosis by Madrid Classification was the ‘lepromatous’ (LL) form in 12 patients, ‘borderline’ (BL) in 2, ‘tuberculoid’ (TT) in 1 patient and ‘indeterminate’ (I) in 1 patient (Additional File 1, Table S1). Most patients had received 2–3 courses of dapsone monotherapy during the 1960s to 1980s; seven had received multidrug therapy (MDT, comprising dapsone, rifampicin, and clofazimine). Type 1 and 2 HD reactions before, during or after HD treatment were recorded for 10 patients.

#### Current HD patients

Of the 21 current HD patients, 9 were female and 12 male, median age 54 (range 33–68) years, age at diagnosis 50 (32–68) years and time since diagnosis 4 (0–15) years. At time of diagnosis, 9 were classified as LL, 5 BL (3 paucibacillary, 2 multibacillary), and 7 TT. All patients received WHO-MDT (dapsone, rifampicin, clofazimine), 14 for MB and 6 for PD HD; one patient was on an alternative regimen. Type 1 and type 2 HD reactions before, during or after Hansen disease treatment were recorded in 17 patients, and were treated using prednisone, thalidomide, and non-steroidal anti-inflammatory drugs (Additional File 1, Table S2).

#### Non-HD controls

The 37 non-HD controls were attending HUCAM for reasons other than HD requiring facial CT scan (Additional File 1, Table S3). The ages of controls (median 62, IQR 57–70, range 33–92 years) matched that of all cases combined (median 62, IQR 58–70, range 33–89 years), as did the numbers of male (18) and female (19) patients (Additional File 1, Table S3). CT scans were requested mostly by otolaryngologists (19/37), followed by general practitioners (10) and head and neck surgeons (4). Sinusitis (of which 11 chronic) or rhinitis was the primary indication in 16 cases, including three cases with immunodeficiency/immunosuppression (one chemotherapy, one thymectomy, one renal transplant). Five patients were referred for reasons related to diagnosis of post-treatment follow-up of carcinomas. There were not trauma cases.

### Maxillofacial bone alterations

There was strong evidence of differences between all HD patients (current and former combined) and non-HD controls only in alteration of the anterior alveolar process of maxilla, which showed severe resorption/atrophy in half (8/16) of former patients, 28.6% (6/21) of current patients, but only 10.8% (4/37) of controls (*p* = 0.003) (Table 1). For the posterior alveolar process of maxilla, evidence was much weaker, with 43.8% (7/16) of former and 47.6% (10/21) of current patients having severe resorption/atrophy, compared with 27.0% (10/37) of controls (*p* = 0.09).

**Table 1:** Maxillofacial bone alterations in current and former Hansen’s disease patients and controls.

Feature	Alteration^†^	Controls (n = 37)	Current patients (n = 21)	Former patients (n = 16)	*p* value^‡^ (controls vs. all patients)	*p* value^‡^ (controls vs. recent vs. former patients)	*p* value^‡^ (recent vs. former patients)
Osteitis	No	33 (89.2%)	18 (85.7%)	14 (87.5%)	1.000	0.901	1.000
	Yes	4 (10.8%)	3 (14.3%)	2 (12.5%)			
Nasal bones	None	21 (56.8%)	15 (71.4%)	6 (37.5%)	0.223	0.012	0.012
	Mild-to-moderate	15 (40.5%)	6 (28.6%)	5 (31.3%)			
	Severe	1 (2.7%)	0 (0.0%)	5 (31.3%)			
Anterior nasal spine	None	21 (56.8%)	12 (57.1%)	4 (25.0%)	0.211	0.069	0.070
	Mild-to-moderate	13 (35.1%)	7 (33.3%)	6 (37.5%)			
	Severe	3 (8.1%)	2 (9.5%)	6 (37.5%)			
Anterior nasal aperture	None	22 (59.5%)	13 (61.9%)	6 (37.5%)	0.488	0.044	0.018
	Mild-to-moderate	13 (35.1%)	8 (38.1%)	5 (31.3%)			
	Severe	2 (5.4%)	0 (0.0%)	5 (31.3%)			
Nasal septum	None	36 (97.3%)	20 (95.2%)	12 (75.0%)	0.231	0.020	0.072
	Mild-to-moderate	0 (0.0%)	1 (4.8%)	1 (6.3%)			
	Severe	1 (2.7%)	0 (0.0%)	3 (18.8%)			
Inferior nasal turbinates	None	20 (54.1%)	11 (52.4%)	8 (50.0%)	0.781	0.800	0.722
	Mild-to-moderate	15 (40.5%)	7 (33.3%)	7 (43.8%)			
	Severe	2 (5.4%)	3 (14.3%)	1 (6.3%)			
Middle nasal turbinates	None	26 (70.3%)	16 (76.2%)	9 (56.3%)	0.403	0.370	0.408
	Mild-to-moderate	10 (27.0%)	3 (14.3%)	5 (31.3%)			
	Severe	1 (2.7%)	2 (9.5%)	2 (12.5%)			
Hard palate	Normal	16 (43.2%)	12 (57.1%)	8 (50.0%)	0.414	0.592	0.746
	Abnormal	21 (56.8%)	9 (42.9%)	8 (50.0%)			
Alveolar process of maxilla (anterior)	None	18 (48.7%)	6 (28.6%)	0 (0.0%)	0.003	0.001	0.051
	Mild-to-moderate	15 (40.5%)	9 (42.9%)	8 (50.0%)			
	Severe	4 (10.8%)	6 (28.6%)	8 (50.0%)			
Alveolar process of maxilla (posterior)	None	11 (29.7%)	2 (9.5%)	2 (12.5%)	0.093	0.299	1.000
	Mild-to-moderate	16 (43.2%)	9 (42.9%)	7 (43.8%)			
	Severe	10 (27.0%)	10 (47.6%)	7 (43.8%)			
Maxillofacial score^¶^	median (IQR)	5 (2–7)	5 (3–7)	7 (5.5–10.5)	0.036	0.010	0.024

^†^Degree of resorption/atrophy for all features except osteitis (present/absent) and hard palate (normal/thinning with bony discontinuities).

^‡^Fisher’s exact (categorical variables) or Kruskal–Wallis test (maxillofacial score).

^¶^A maxillofacial bone alteration score was calculated as a sum of the score for each feature, where no change was scored 0, mild-to-moderate alterations 1, and severe 2. This yielded a theoretical range of 0–18.

Evidence of differences across the three groups was determined mainly by severe resorption/atrophy being more frequent among former patients compared with current patients and controls. Severe resorption/atrophy of nasal bones was observed in 31.3% (5/16) of former compared with no current patients and one control (*p* = 0.01), the nasal aperture had severe alterations in 31.3% (5/16) of former compared with no current patients and two controls (*p* = 0.04), and the nasal septum had severe or mild-to-moderate alterations in 25.0% (4/16) of former compared with one current patient and one control (*p* = 0.02).

**Figure 1:**
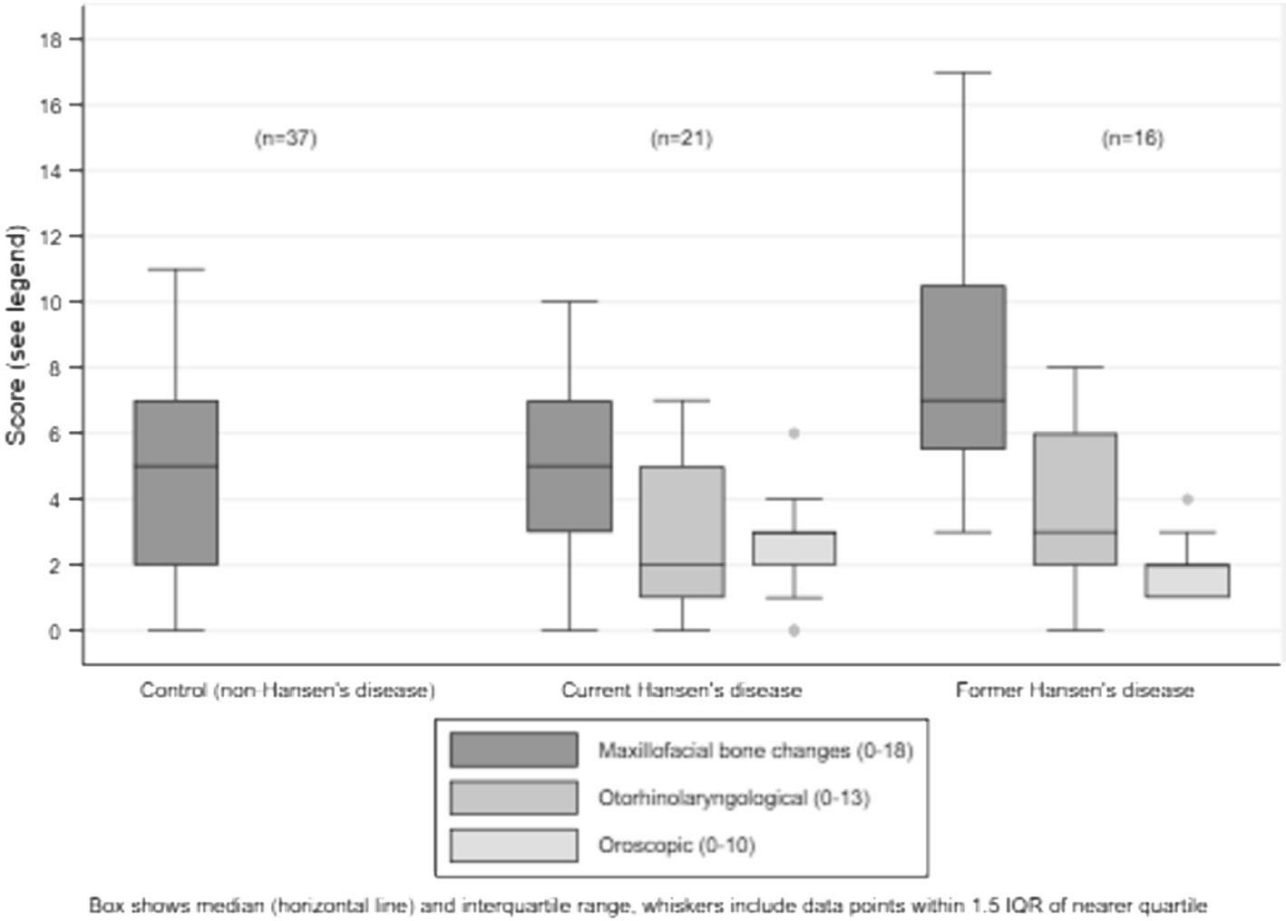
Maxillofacial bone change score and otorhinolaryngological and oroscopic finding scores in controls and in current and former Hansen’s disease patients.

Maxillofacial bone alteration score was higher in former HD patients (median 7, IQR 5.5–10.5) than in current patients (median 5, IQR 3–7) and controls (median 5, IQR 2–7) (Fig. 1). Although current HD patients had higher prevalence, compared with controls, of alterations to nasal turbinates and alveolar processes of maxilla, these differences were not supported by statistical evidence and median maxillofacial score was the same in both groups (*p* = 0.544).

Severe alterations in control patients included one case presenting with sequelae of mucocutaneous leishmaniasis comprising nasopharyngeal fibrosis, absence of uvula, nasal septal perforation, and nasal tip collapse, one case of previously operated squamous cell carcinoma of the face, and one case of basal cell carcinoma of the right orbit submitted to exenteration and complementary radiotherapy. Three control patients presented severe resorption of the anterior nasal spine, one patient with systemic lupus erythematosus (SLE) and two patients with a history of chronic sinusitis. Four control patients had severe resorption of the alveolar process of maxilla (anterior), three of whom were missing all 16 maxillary teeth, one missing 13 maxillary teeth including the 4 central incisors. Severe resorption of the alveolar process of maxilla (posterior) was observed in 10 control patients, of whom seven had lost all 16 maxillary teeth and the other three had lost 10–13 maxillary teeth. Twenty-one control patients had thinning of the hard palate with bony discontinuities, possibly attributable to osteoporosis and/or tooth loss (11/21 were missing all 16 upper teeth, five were missing 8–13 maxillary teeth, and the remaining five were missing 1–6 maxillary teeth).

### Otorhinolaryngological and oroscopic findings

Findings from rhinoscopic, nasal endoscopic and oroscopic examinations, which were performed only in former and current HD patients, are summarized in Tables 2 and 3. Of the nasal features shown in Table 2, former HD patients generally presented with more abnormalities, but the sample size was not big enough to detect an actual difference. The only differences support by statistical evidence were in the colour of the oral mucosa, which was normal for all former patients but pale or hyperaemic for half (10/21) of the current patients (*p* = 0.002), and loss of upper incisors, for which all former patients had lost 3 or 4 teeth compared with three-quarters of current patients (*p* = 0.02) (Table 3).

**Table 2:** Rhinoscopy and nasal endoscopy findings in current and former Hansen’s disease patients.

Feature	Finding	Current patients (n = 21)	Former patients (n = 16)	*p* value (Fisher’s exact)
Nasal floor (integrity)	Normal	21 (100.0%)	16 (100.0%)	–
	Perforated	0 (0.0%)	0 (0.0%)	
Nasal floor (crusting)	Not crusted	18 (85.7%)	11 (68.8%)	0.254
	Crusted	3 (14.3%)	5 (31.2%)	
Nasal floor (secretion)	None	19 (90.5%)	15 (93.8%)	1.000
	Hyaline	0 (0.0%)	0 (0.0%)	
	Mucous	2 (9.5%)	1 (6.3%)	
	Purulent	0 (0.0%)	0 (0.0%)	
Nasal floor (polyps)	None	21 (100.0%)	16 (100.0%)	–
	Present	0 (0.0%)	0 (0.0%)	
Nasal septum	Centred	11 (52.4%)	9 (56.3%)	0.304
	Deviation 1–3	8 (38.1%)	3 (18.7%)	
	Deviation 4–5	1 (4.8%)	0 (0.0%)	
	Perforated 1–3	1 (4.8%)	3 (18.7%)	
	Perforated 4–5	0 (0.0%)	1 (6.3%)	
Inferior nasal turbinates (trophism)	Normotrophic	12 (57.1%)	7 (43.8%)	0.165
	Hypertrophic	3 (14.3%)	0 (0.0%)	
	Atrophic	6 (28.6%)	9 (56.3%)	
Inferior nasal turbinates (colour)	Normal	11 (52.4%)	7 (43.8%)	0.868
	Pale	9 (42.9%)	8 (50.0%)	
	Hyperaemic	1 (4.8%)	1 (6.3%)	
Inferior nasal turbinates (crust or polyp)	None	18 (85.7%)	12 (75.0%)	0.437
	Crust	3 (14.3%)	4 (25.0%)	
	Polyp	0 (0.0%)	0 (0.0%)	
Middle nasal turbinates (trophism)	Normotrophic	15 (71.4%)	8 (50.0%)	0.165
	Hypertrophic	1 (4.8%)	0 (0.0%)	
	Atrophic	5 (23.8%)	8 (50.0%)	
Middle nasal turbinates (colour)	Normal	11 (52.4%)	7 (43.8%)	0.868
	Pale	9 (42.9%)	8 (50.0%)	
	Hyperaemic	1 (4.8%)	1 (6.3%)	
Middle nasal turbinates (crust or polyp)	None	19 (90.5%)	14 (87.5%)	0.758
	Crust	2 (9.5%)	1 (6.3%)	
	Polyp	0 (0.0%)	1 (6.3%)	
Inferior meatus	Free	16 (76.2%)	13 (81.2%)	0.681
	Crust	3 (14.3%)	3 (18.7%)	
	Secretion	2 (9.5%)	0 (0.0%)	
	Polyp	0 (0.0%)	0 (0.0%)	
Middle meatus	Free	20 (95.2%)	13 (81.2%)	0.494
	Crust	1 (4.8%)	1 (6.3%)	
	Secretion	0 (0.0%)	1 (6.3%)	
	Polyp	0 (0.0%)	1 (6.3%)

**Table 3:** Oroscopic findings in current and former Hansen’s disease patients.

Feature	Finding	Current patients (n = 21)	Former patients (n = 16)	*p* value^‡^
Oral mucosa	Normal	11 (52.4%)	16 (100.0%)	0.002
	Pale	8 (38.1%)	0 (0.0%)	
	Hyperaemic	2 (9.5%)	0 (0.0%)	
Palate	Normal	18 (85.7%)	15 (93.8%)	0.618
	Ogival	3 (14.3%)	1 (6.2%)	
	Medium hard perforation	0 (0.0%)	0 (0.0%)	
	Paramedium hard perforation	0 (0.0%)	0 (0.0%)	
	Medium soft perforation	0 (0.0%)	0 (0.0%)	
	Paramedium soft perforation	0 (0.0%)	0 (0.0%)	
Mouth (Hansen’s disease nodules)	None	21 (100.0%)	16 (100.0%)	–
	Present	0 (0.0%)	0 (0.0%)	
Oropharynx (Hansen’s disease nodules)	None	21 (100.0%)	16 (100.0%)	–
	Present	0 (0.0%)	0 (0.0%)	
Tongue	Normotrophic	17 (81.0%)	15 (93.8%)	0.780
	Atrophic	1 (4.8%)	0 (0.0%)	
	Geographic	3 (14.3%)	1 (6.2%)	
Uvula	Normal	19 (90.5%)	15 (93.8%)	1.000
	Bifid	0 (0.0%)	0 (0.0%)	
	Elongated	2 (9.5%)	1 (6.2%)	
Amygdala	I/II	18 (85.7%)	16 (100.0%)	0.243
	III/IV	3 (14.3%)	0 (0.0%)	
	Tonsillectomy	0 (0.0%)	0 (0.0%)	
Tonsil pillar (colour)	Normal	15 (71.4%)	15 (93.8%)	0.294
	Pale	2 (9.5%)	0 (0.0%)	
	Hyperaemic	4 (19.1%)	1 (6.2%)	
Dental disease	None	12 (57.1%)	6 (37.5%)	0.325
	Present	9 (42.9%)	10 (62.5%)	
Upper incisors	All present	5 (23.8%)	0 (0.0%)	0.024
	Loss of 1	0 (0.0%)	0 (0.0%)	
	Loss of 2	0 (0.0%)	0 (0.0%)	
	Loss of 3	0 (0.0%)	3 (18.7%)	
	Loss of 4	16 (76.2%)	13 (81.3%)	
Number of upper decayed teeth^†^	Median (IQR)	0 (0–4)	0 (0–0)	0.481
Number of missing upper teeth^†^	Median (IQR)	16 (12–16)	16 (16–16)	0.453

^‡^Fisher’s exact (categorical variables) or Kruskal–Wallis test (number of decayed or missing upper teeth).

^†^Not included in oroscopic examination score—all other features included, scored as 0 for none/normal, 1 for any other finding.

Dental data (numbers of missing upper teeth and upper incisors) were available for former and current HD patients and non-HD controls. There were no differences between former and current patients (Table 3), but controls had less tooth loss (median number of missing upper teeth 12, IQR 4–16, *p* = 0.008 compared with HD patients (current and former combined, median 16, IQR 14–16). In controls, 41.7% (15/36) had all upper incisors and 50.0% (18/36) had lost four, compared with 13.5% (5/37) of HD patients with all upper incisors present and 78.4% (29/37) with four lost (*p* = 0.002).

### Correlation between otorhinolaryngological and oroscopic findings and maxillofacial bone alterations in current and former HD patients

**Figure 2:**
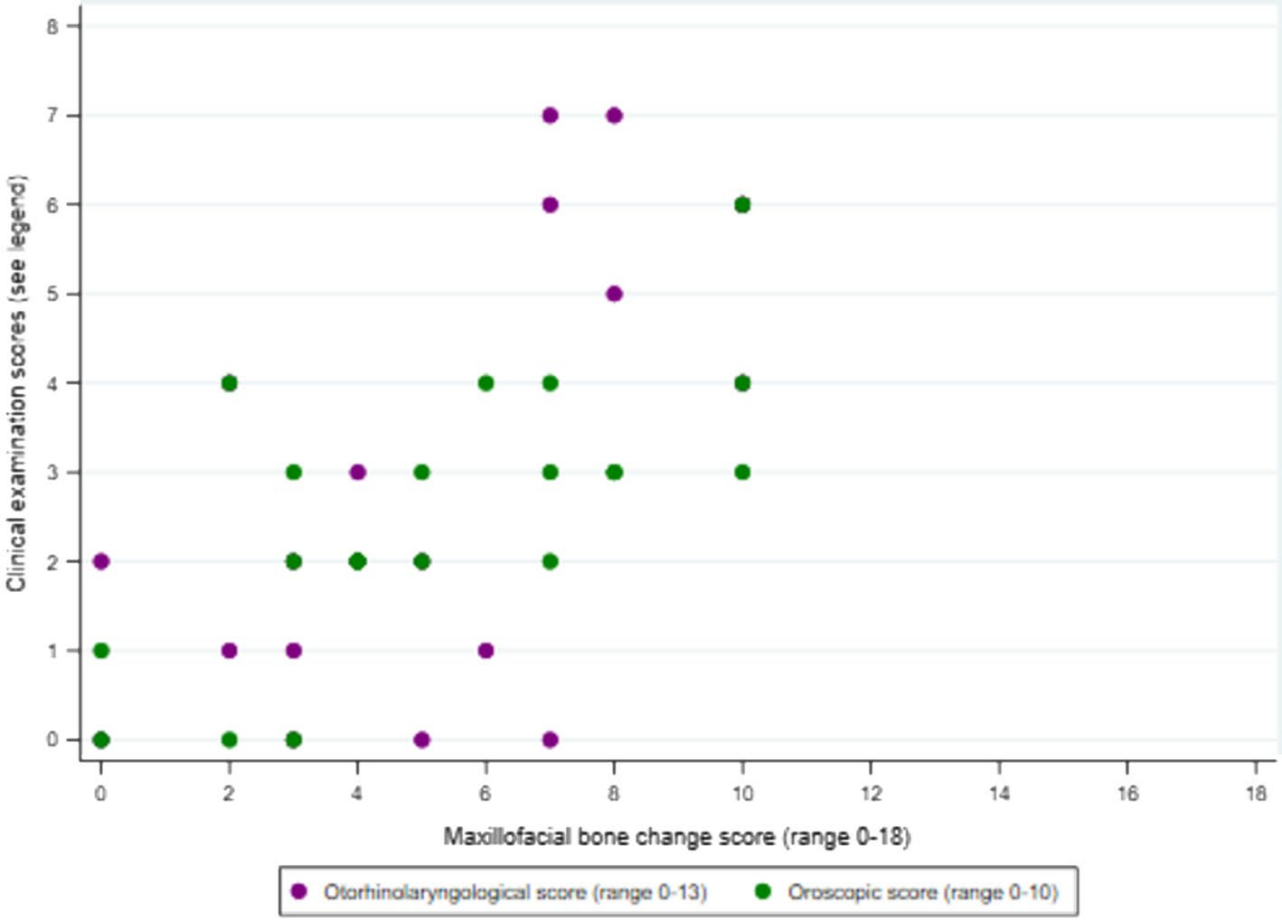
Correlation between otorhinolaryngological and oroscopic findings and maxillofacial bone alterations in current Hansen’s disease patients (see Table 4 for correlation coefficients).

**Figure 3:**
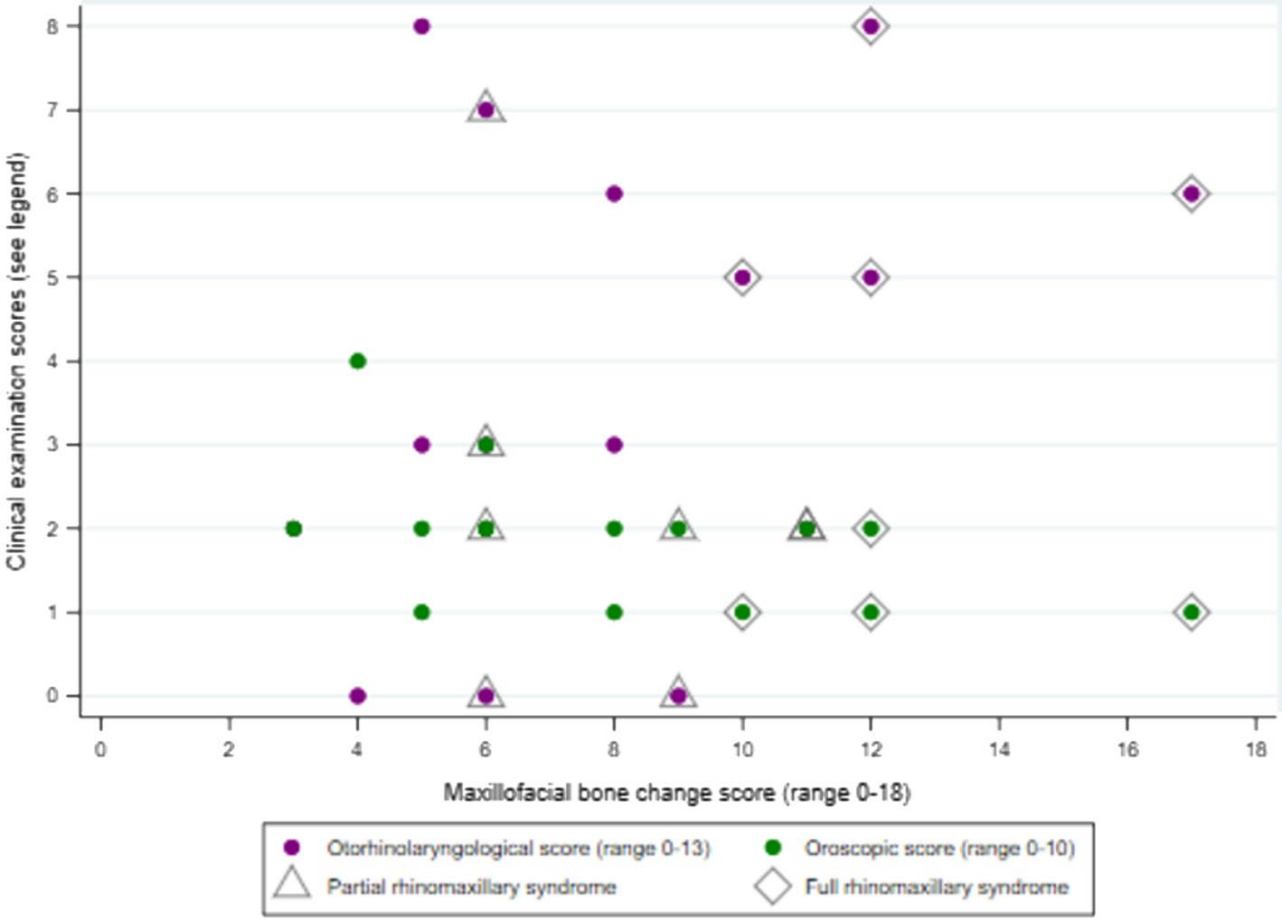
Correlation between otorhinolaryngological and oroscopic findings and maxillofacial bone alterations in former Hansen’s disease patient (see Table 4 for correlation coefficients).

Median maxillofacial score was higher in former compared with current patients (*p* = 0.02) whereas otorhinolaryngological and oroscopic scores were similar (Table 4, Fig. 1). In current HD patients, there was strong positive correlation between the three scores, with Pearson’s correlation coefficients ≥ 0.6 (all *p* ≤ 0.004) (Table 4, Fig. 2). In former HD patients, absence of correlation appeared to be a consequence of a wider range of maxillofacial scores and a narrower distribution of oroscopic scores (Table 4, Fig. 3). There was only weak evidence of an inverse correlation between oroscopic and maxillofacial scores (coefficient -0.5, *p* = 0.05), determined mainly by one patient with RMS who had a high maxillofacial score but low oroscopic score and two patients with lower maxillofacial scores who had higher oroscopic scores (Fig. 3).

**Table 4:** Correlation between otorhinolaryngological and oroscopic findings and maxillofacial bone alterations in current and former HD patients.

Score	Patient group	Median (IQR)	*p* value^‡^	Correlation with maxillofacial score (*p* value)	Correlation with oroscopic score (*p* value)	Correlation with age (*p* value)
**Current versus former HD patients**						
Maxillofacial (range 0–18)	Current (n = 21)	5 (3–7)	0.024	–	–	0.144 (0.534)
	Former (n = 16)	7 (6–11)		–	–	0.469 (0.067)
Otorhinolaryngological (range 0–13)	Current (n = 21)	2 (1–5)	0.321	0.646 (0.002)	0.590 (0.005)	0.150 (0.517)
	Former (n = 16)	3 (2–6)		0.364 (0.166)	− 0.377 (0.151)	0.716 (0.002)
Oroscopic (range 0–10)	Current (n = 21)	3 (2–3)	0.083	0.698 (< 0.001)	–	0.422 (0.057)
	Former (n = 16)	2 (1–2)		− 0.496 (0.051)	–	− 0.467 (0.068)
**Multibacillary (MB) versus paucibacillary (PB) HD**						
Maxillofacial (range 0–18)	PB (n = 13)	4 (2–5)	0.002	–	–	0.427 (0.146)
	MB (n = 24)	8 (6–10)		–	–	0.329 (0.117)
Otorhinolaryngological (range 0–13)	PB (n = 13)	2 (0–2)	0.002	0.057 (0.852)	0.151 (0.623)	0.335 (0.264)
	MB (n = 24)	5 (2–6)		0.348 (0.095)	0.256 (0.226)	0.266 (0.208)
Oroscopic (range 0–10)	PB (n = 13)	2 (2–3)	0.886	0.548 (0.052)	–	0.429 (0.153)
	MB (n = 24)	2 (1.5–3)		0.027 (0.901)	–	− 0.319 (0.129)
**Male versus female**						
Maxillofacial (range 0–18)	Female (n = 19)	5 (4–8)	0.475	–	–	0.294 (0.221)
	Male (n = 19)	7 (4–10)		–	–	0.571 (0.013)
Otorhinolaryngological (range 0–13)	Female (n = 19)	2 (0–3)	0.056	0.395 (0.014)	0.534 (0.001)	0.218 (0.371)
	Male (n = 19)	4.5 (2–6)		0.507 (0.002)	0.581 (0.002)	0.554 (0.017)
Oroscopic (range 0–10)	Female (n = 19)	2 (2–3)	0.670	0.233 (0.159)	–	0.046 (0.851)
	Male (n = 19)	2 (1–3)		0.393 (0.018)	–	− 0.059 (0.816)

^‡^Kruskal–Wallis test comparing current versus former or MB versus PB or male versus female.

### Correlations of maxillofacial bone alterations and otorhinolaryngological and oroscopic findings with demographic and clinical characteristics of current and former HD patients

**Figure 4:**
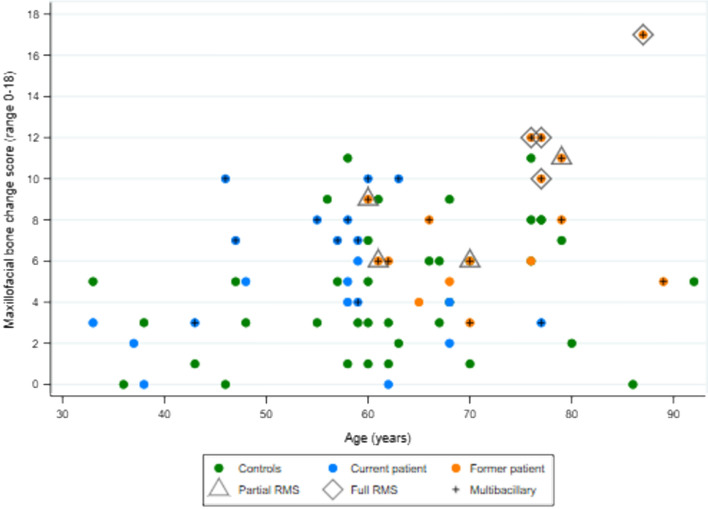
Correlation between maxillofacial bone alterations score and age in current and former Hansen’s disease patients and non-Hansen’s disease controls (see Table 4 for correlation coefficients).

**Figure 5:**
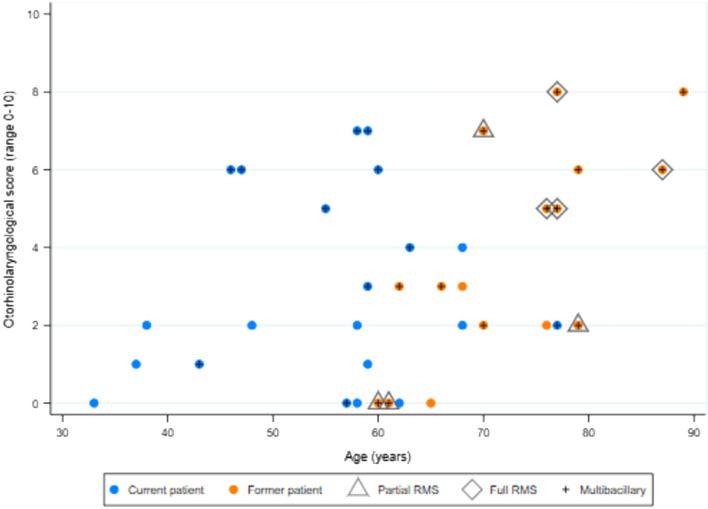
Correlation between otorhinolaryngological score and age in current and former Hansen’s disease patients (see Table 4 for correlation coefficients).

**Figure 6:**
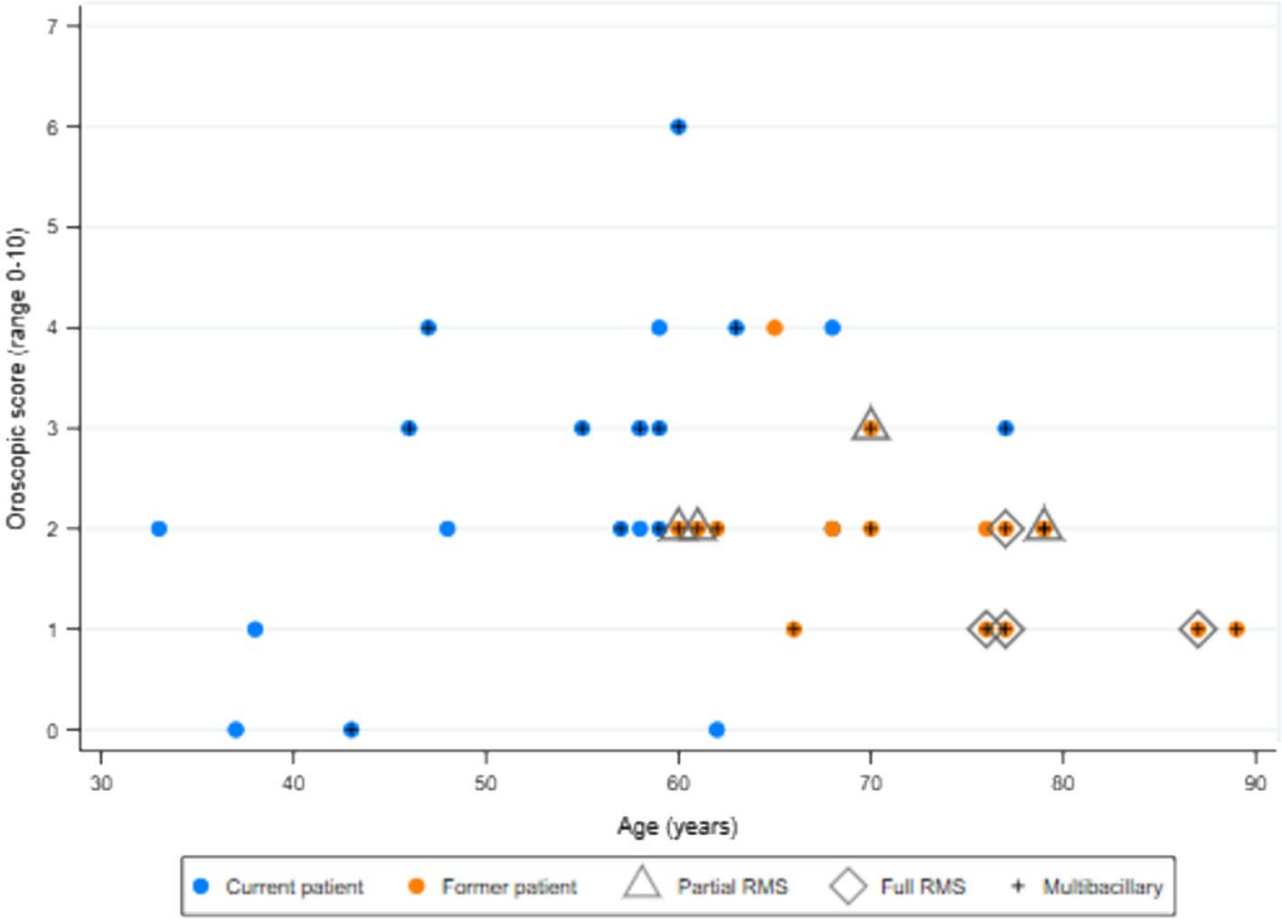
Correlation between oroscopic score and age in current and former Hansen’s disease patients (see Table 4 for correlation coefficients).

Maxillofacial score was modestly correlated with age (coefficient 0.368, *p* = 0.001) across all groups combined (controls and patients, Fig. 4) and among all patients (coefficient 0.452, *p* = 0.005) but, in subgroups, was correlated with age only among male patients (current and former) (0.571, *p* = 0.013) not with female patients or in subgroups comprising PB or MB patients (Table 4). Otorhinolaryngological score was modestly correlated with age (coefficient 0.384, *p* = 0.019) across both patient groups combined (Fig. 5). In subgroups, it was correlated with age among former patients (coefficient 0.716, *p* = 0.002) and among male patients (coefficient 0.554, *p* = 0.017) but not among female patients or in subgroups comprising PB or MB patients. Oroscopic score was not correlated with age across the combined patient groups (Fig. 6) or in any subgroups.

### Rhinomaxillary syndrome (RMS)

Full RMS was diagnosed in 4 former patients, partial RMS in 4, and no RMS in the other 8 former patients. These patients have been described previously^13^; their clinical histories are summarized in Table S1 (Additional File 1), maxillofacial alterations in Table S4, and otorhinolaryngological and oroscopic findings in Tables S5 and S6 (Additional File 1). Although the sample size was small, some of the expected differences in maxillofacial bone alterations were statistically evident (Additional File 1, Table S4) as was a higher proportion of nasal septum perforation in former patients with RMS (Additional File 1, Table S5). The median maxillofacial score was 12 in full RMS cases, 7.5 in partial RMS and 5.5 in absence of RMS (*p* = 0.007). There was no difference in otorhinolaryngological and oroscopic scores (Additional File 1, Tables S5 and S6).

## Discussion

### Maxillofacial bone alterations

Our investigation of maxillofacial bone manifestations of HD in former HD patients compared with current HD patients and non-HD controls found that differences in bone alterations across the three groups were determined mainly by severe resorption/atrophy being more frequent in former HD patients, with the anterior alveolar process of maxilla showing severe resorption/atrophy in 50% of former patients, 29% of current patients and 11% of controls, nasal bones and aperture showing severe resorption/atrophy in 31% of former patients compared with no current patients and one/two controls, and the nasal septum severe or mild-to-moderate alterations in 25% of former HD patients compared with one current patient and one control. These findings are consistent with most of the former HD patients having been diagnosed and treated in the pre-MDT era and with much longer time between initial treatment and the present day allowing for greater disease progression.

Current HD patients had a higher prevalence, compared with controls, of alterations to nasal turbinates and alveolar processes of maxilla. That these differences were not supported by statistical evidence can be attributed to small sample size. These alterations merit attention because they serve as early indication of progression towards the more severe changes observed in former HD patients. Even if arrested by the WHO-MDT that the current HD patients in our study were receiving, these alterations are irreversible and could become a source of secondary complications.

That HD-related bone degradation might continue after a patient has been ‘cured’, particularly were cure is determined by the end of a standard fixed-duration regimen and where HD reactions have occurred^15,16^, is a possibility supported by evidence for continuation of bone resorption post-treatment in a study from Brazil showing partial or total loss of one or more phalanges or metatarsal/carpal bones in 18% (19/105) of patients who had repeat radiological examinations up to 8 years post-MDT^17^. Whilst macroscopic bone alterations caused by *M. leprae* infection are reasonably well-characterised in clinical studies^17–19^, the processes underlying HD-related bone changes and associations of these changes with circulating concentrations of bone minerals and related metabolites are poorly understood^20^. In recently-diagnosed HD patients, bone mineral density measured at femoral neck and hip was lower in patients than in controls^21^. Other studies have reported HD-related differences in physiological levels of bone minerals and metabolites, including LL HD patients having lower total calcium, higher alkaline phosphatase and higher urinary hydroxyproline than BL HD patients^22^. These small studies have yet to be replicated. By contrast, resorption secondary to HD-related neuropathy is well-documented. A 2001 study in the USA based on radiological examination mainly of bones of the hands and feet found no differences in bone alterations comparing patients admitted to Carville hospital in the early 1900s (before chemotherapy) with the last patients still under care (most of whom were admitted and treated within the first year of two of diagnosis)^23^. The authors of this study interpreted most of the observed changes as being trauma-related and secondary to nerve impairment, suggesting that patients in the treatment era either presented too late to prevent permanent nerve damage or that treatment needed to be followed by long-term care including regular examination for injury. Nasal alterations and other HD-related impairments, including sensory loss and osteoarticular changes to the hands^8^, can lead to secondary complications as a result of impeded nasal hygiene^12^.

### Otorhinolaryngological and oroscopic findings

Given that severe maxillofacial bone alterations were more common in former compared with current HD patients, the absence of major differences in otorhinolaryngological and oroscopic findings between the two patient groups is perhaps surprising. We had previously shown that the more severe forms of maxillofacial bone alteration (RMS) in the former HD patient group correlated with visible facial profile changes, including saddle nose, concave middle third of face with sunken nose, inverted upper lip and maxillary retrognathia^13^. Former HD patients did tend to present with more otorhinolaryngological abnormalities than current HD patients, but our study had low power to detect differences between these two groups, and we would need to take into account the younger age of the current HD patient group.

Similarly, we might have expected correlation between our scores for maxillofacial, otorhinolaryngological and oroscopic findings in both patient groups, but this was evident only in current HD patients. The strong correlation between otorhinolaryngological and maxillofacial bone alteration scores indicates that the former might be a useful clinical proxy for the latter, suitably adjusted for age and sex, supporting our proposal that clinical protocols for HD patient assessment and follow-up be extended to include otorhinolaryngological evaluation, supported by radiological imaging where necessary, to assess disease progression.

Interestingly, whilst there was no difference in tooth loss between current and former patients, as might be expected on the basis of older age, we found that HD patients had more tooth loss than the age-matched control group. Whilst this difference might be confounded by unmeasured social and other health factors^24^, a causal association is also possible. Tooth loss, the primary cause of which is dental disease, typically leads to resorption of the alveolar process of the maxilla and might cause hard palate thinning. Therefore, it is not possible to attribute resorption of the alveolar process of the maxilla only to Hansen's disease. Indeed, all of the control patients in our study who had severe resorption of the anterior or posterior alveolar process had lost 10 or more maxillary teeth. Kasai et al. reported that HD-related changes occur predominantly in the median frontal part with characteristic interruption of the U-shaped process^25^, as we also observed in our patients, compared with the typical horizontal and vertical age-related atrophy of the maxilla and mandible^26,27^. Given that our cases and controls were age-matched, *M. leprae* is commonly present in oral mucosa^28^, and the interplay between HD and oral health is poorly understood, a possible causal role of HD in tooth loss cannot be discounted^29^. That we found as many control as HD patients with hard palate bone thinning and discontinuities could be related to bone demineralization (osteoporosis) and/or tooth loss in all groups in our study^30,31^. However, spatial resolution presents a limitation to CT assessment of fine structures such as the hard palate (if the thickness of the hard palate is less than the spatial resolution) therefore characterisation of this particular feature in our study is susceptible to random misclassification.

### Strengths and weaknesses

A strength of our study is that it included HD patients and non-HD controls from the same population area. Although matched to ensure that the combined patient group and controls had similar age and sex distributions, other potentially important differences, such as socioeconomic and other health factors, may have existed between the groups. We also made comparisons between current and former HD patients which were two very different groups in terms of age, period when diagnosed and treatment history. A major limitation of our study is its small size, meaning that our findings require replication in a larger study. We had incomplete clinical histories for some former HD patients because records were lost over time at the hospital. Resorption and atrophy of the nasal bones can be a consequence of trauma and surgery, which may not have been recorded in patients’ medical histories. One former patient developed nasal myiasis at a later date (after the CT scan in this study)^12^. Similarly, we did not have complete medical records for former HD patients to support our assessment of differential diagnoses such as syphilis and mucocutaneous leishmaniasis (as observed in one control patient)^14^, and less common rhinological diseases such as granulomatosis with polyangiitis and nasal extranodal lymphoma^32^. Medical records for current patients were more complete and none indicated previous surgical interventions. Bone alterations observed in control patients had a range of possible and known causes, the latter including sequelae of mucocutaneous leishmaniasis, carcinomas, SLE, and chronic sinusitis. SLE may present with nasal involvement with a spectrum of symptoms, mucosal changes and perforation of the nasal septum secondary to vasculitis or ischemia with subsequent chondrolysis^33,34^. Our data did not include aetiologies of chronic sinusitis in control patients, but allergic fungal sinusitis has been known to cause erosion of the bony sinus walls^35,36^.

### Implications and suggestions for future research

Our description of maxillofacial bone alterations in older persons affected by HD (former HD patients) adds to an existing body of evidence. Elderly patients are susceptible to poor health and incapacity in general, and these age-related processes will combine with lifelong effects of HD to reduce personal autonomy and increase the risk of secondary health problems^12,37^. Our finding of correlation between maxillofacial bone alterations and otorhinolaryngological manifestations in current HD patients is novel and merits replication in a larger study. Clinical and public health interventions which minimise the development of irreversible changes will reduce the substantial burden of HD-related disability and stigmatizing manifestations of HD in endemic countries^38^. In the meantime, this finding provides further evidence that HD patient management should include a full otorhinolaryngological evaluation, as a minimum, supported by radiological imaging where necessary^11,13^. Our findings also show that dental and oral health in persons affected by HD should not be neglected as part of assessment and care.

Finally, global burden of disease estimates for HD are grossly under-estimated because disability weight calculations for HD assign zero weight to persons affected by HD who do not have any severe disability (grade 0), and low weights to grade 1 and 2 disability (0.01 and 0.07, respectively)^39^. Nanjan Chandran et al*.* calculated revised weights by including skin lesions, mild pain and mental distress (but no visible disfigurement) in the grade 0 health state descriptor for HD, and greater emphasis on mental distress and difficulty with social and daily activities in the grade 1 and 2 descriptors. Whilst the correspondingly higher weights will yield more valid disease burden estimates for HD, we would propose that consideration should also be given to otorhinolaryngological and orodental manifestations of HD and to ‘invisible’ maxillofacial bone alterations which are precursors of visible changes^11,13^.

## Conclusion

Far from being a disease of antiquity, Hansen’s disease still represents a major challenge for public health systems in endemic countries, where resources and expertise are often limited. Our findings require replication in a larger study. In the meantime, otorhinolaryngological and dental evaluations are low cost and potentially preventive measures that could be incorporated into clinical guidelines for patients presenting as new cases, under treatment or during follow-up. Impacts of otorhinolaryngological and orodental manifestations of HD on patient quality-of-life should be considered for inclusion in health state descriptors for HD disability weights.

## Supplementary Information

Supplementary Tables.

## Data Availability

The datasets analysed during the current study are not publicly available due to patient confidentiality but are available from the corresponding author on reasonable request.
