# Supplementary material for: Clinical and radiological evaluation of maxillofacial and otorhinolaryngological manifestations of Hansen’s disease
Source: Sci Rep. 2022 Sep 1;12:14912. doi: 10.1038/s41598-022-19072-0 (PMC9436959; doi:10.1038/s41598-022-19072-0)
Supplement: Supplementary file 1 — Supplementary Tables. [file 41598_2022_19072_MOESM1_ESM.pdf]

Table S1. Former Hansen's disease patients (n=16): diagnosis, treatment history and reactions

ID	Age	Sex	Madrid classification [a]	Year of diagnosis	Age at diagnosis	Delay (months) [b]	Mono-therapy start year (drug) [c]	Months of mono-therapy (drug) [c]	Multidrug therapy	Hansen's disease reactions [d]			Reaction therapy [e]	Rhino-maxillary syndrome (RMS)
										Before	During	After		
P1	87	M	LL	1970	42		1970 (D)	144 (D)	yes	-	II	-	P, T	Full
P2	77	M	LL	1964	27		1977 (D)	156 (D)	none	-	-	III	P	Full
P3	89	M	LL	1968	43		1978 (D)		none	-	-	II	P, T	No
P4	65	F	TT		20	120				-	-	I	P	No
P5	62	F	LL		25	2	1978 (D)<br>1990 (R)		none	-	II	II	P, T	No
P6	68	F	BL		13		1975 (D)		none	-	-	II	T	No
P7	77	F	LL		31	60	1975 (C)<br>1979 (R)<br>1983 (D)	300 (D)	yes	II	II	-	T	Full
P8	76	M	LL		6	12	1969 (D)<br>1984 (C)<br>1984 (R)	252 (D)	yes	-	-	-		Full
P9	79	F	LL	1949	9		1960 (D)<br>1984 (C)<br>1990 (D)		none	-	-	-		Partial
P10	79	F	LL	1959	11		1959 (D)<br>1975 (C)<br>1979 (R)		yes	-	-	-		No
P11	60	F	LL	1976	17	3	1976 (D)<br>1979 (R)	180 (D) 36 (R)	yes	-	-	-		Partial
P12[f]	70	F	LL		17	96				-	-	I	P	No

ID	Age	Sex	Madrid classification [a]	Year of diagnosis	Age at diagnosis	Delay (months) [b]	Mono-therapy start year (drug) [c]	Months of mono-therapy (drug) [c]	Multidrug therapy	Hansen's disease reactions [d]			Reaction therapy [e]	Rhino-maxillary syndrome (RMS)
										Before	During	After		
P13	70	M	LL		26		1963 (D)<br>1984 (R)<br>1988 (C)	300 (D) 60 (R) 36 (C)	yes	-	-	-	-	Partial
P14	76	F	I		23		1968 (D)<br>1984 (C)<br>1987 (R)	60 (D) n/r (C)<br>24 (R)	yes	-	-	-	-	No
P15	61	F	LL	1970	15	48	1972 (D)<br>1975 (C)<br>1978 (D)	36 (D) 180 (C) 96 (D)	none	-	I<br>I	-	T	Partial
P16	66	M	BL		40	36	1970 (D)<br>1980 (C)	120 (D)	none	II	I<br>I	-	P, T	No

[b] Madrid Classification: LL = 'lepromatous'; BL = 'borderline'; I = 'indeterminate'; TT = 'tuberculoid'

[b] Delay in diagnosis was defined as being the time (in months) from awareness of the first symptom to the start of treatment

[c] Drugs: D = dapsone; R = rifampicin; C = clofazimine

[d] HD reactions: type I ('reversal reaction'); type II (erythema nodosum leprosum); type III (isolated neuritis) - dash indicates no recorded reaction

[e] HD reaction treatments: P = Prednisone; T = Thalidomide.

[f] This person came from Rio de Janeiro therefore data are incomplete

Table S2. Current Hansen's disease patients (n=21): diagnosis, treatment history and reactions

ID	Age	Sex	Madrid classification at diagnosis[a]	Time since diagnosis (years)[b]	Delay (months)[c]	Multidrug therapy[d]	Hansen's disease reactions[e]			HD reaction therapy[f]
							Before	During	After	
P17	61	F	LL	9	9	MB	-	-	-	-
P18	47	F	LL	15	36	MB	-	II	-	T
P19	46	F	LL	3	36	MB	-	II	II	P, T
P20	59	M	LL	4	24	MB	-	-	-	-
P21	37	M	TT	5	12	MB	-	-	-	-
P22	43	F	LL	8	-	MB	-	II	II	P, NSAI, T
P23	58	F	TT	1	24	PB	-	I	-	P, NSAI
P24	55	M	LL	13	2	MB	-	II	II	P, T
P25	59	M	LL	0	60	MB	II	-	-	-
P26	62	M	BL	2	72	MB	I	I	-	P
P27	58	F	BL	1	4	MB	-	I	-	P
P28	58	M	LL	1	2	MB	-	I	-	P, NSAI
P29	57	M	BL	3	8	MB	I	I	I	P
P30	63	M	LL	1	8	AT	-	I	-	P, NSAI
P31	68	M	TT	7	12	PB	-	I	I	P, NSAI
P32	59	M	TT	3	12	PB	-	I	I	P, NSAI
P33	48	F	BL	3	1	MB	-	I	I	P, NSAI
P34	38	M	TT	2	4	PB	-	I	I	P, NSAI
P35	60	M	BL	3	-	MB	-	-	I	NSAI
P36	68	F	TT	0	12	PB	-	-	-	-
P37	33	F	TT	0	12	PB	I	I	-	P

[a] Madrid Classification: LL = 'lepromatous'; BL = 'borderline'; TT = 'tuberculoid'

[b] Time elapsed from the diagnosis of Hansen's disease to the time of the interview (in years)

[c] Delay in diagnosis was defined as the time (in months) from awareness of first symptom(s) to start of treatment

[d] WHO multidrug therapy (MDT): dapsone, rifampicin and clofazimine; alternative (AT)

[e] HD reactions: type I ('reversal reaction'); type II (erythema nodosum leprosum); type III (isolated neuritis) - dash indicates no reaction

[f] HD reaction treatments: P = Prednisone; T = Thalidomide, NSAI = non-steroidal anti-inflammatory

Table S3. Characteristics of non-Hansen's disease patients (control group)

Age	Sex	Signs and symptoms	Specialty	Indication
61	F	Chronic headache	Otolaryngologist	Chronic sinusitis
63	M	Headache	General practitioner	Chronic sinusitis
62	F	Asymptomatic, with no signs of tumor recurrence	Head and Neck Surgeon	Control after left parotidectomy and radiotherapy for parotid myoepithelial carcinoma
58	F	Absence of uvula, nasopharyngeal fibrosis, nasal septal perforation, nasal tip collapse	Otolaryngologist	Sequelae of the bone architecture of the face due to leishmaniasis in the past
38	M	Nasal septum deviation	General practitioner	Allergic rhinitis
47	F	Headache, productive night cough. X-ray of sinuses of the fac: agenesis of the right frontal sinus and hypoplastic left frontal sinus	General practitioner	Chronic pansinusitis
48	F	Hyposmia, dysgeusia	Otolaryngologist	Chronic rhinosinusitis/malignant neoplasm of the upper nasopharyngeal wall
77	F	Hypoacusis in left ear	Otolaryngologist	Left ear mixed hearing loss
43	F	Chronic headache, nasal obstruction	Otolaryngologist	Chronic sinusitis, nasal obstruction
76	F	Headache, nasal obstruction, bilateral tinnitus, bilateral chronic otalgia	Otolaryngologist	Rhinosinusitis/nasal obstruction
46	F	Pain in the maxillary sinus region and nocturnal nasal obstruction	Otolaryngologist	Pansinusitis
36	M	Chronic nasal obstruction, mouth breathing, septal deviation to the right, posterior laryngitis, rhinopathy, sensorineural deafness	Otolaryngologist	Nasal obstruction and nasal septum deviation
62	M	Sinus bleeding, nasal congestion and fever	General practitioner	Acute sinusitis (fungal infection?) and lymphoma in chemotherapy, immunosuppressed
57	F	Recurrent upper airway infection, bronchiectasis	Pulmonologist	Chronic rhinosinusitis, post thymectomy immunodeficiency
67	F	Fetid nasal discharge, dry cough, headache	General practitioner	Chronic sinusitis
55	M	Chronic nasal obstruction, headache, dry cough	Otolaryngologist	Chronic sinusitis
56	M	Recurrent episodes of sinusitis, kidney transplant	Otolaryngologist	Chronic rhinosinusitis, right septum deviation
58	M	Nasal septum deviation, mouth breathing	Otolaryngologist	Nasal septum deviation
59	M	Hypogeusia, anosmia	Otolaryngologist	Chronic sinusitis, allergic rhinitis
70	M	Chronic headache, pain in the malar region of the face biliterally	Otolaryngologist	Chronic sinusitis
70	F	Constant nasal obstruction, headache	Otolaryngologist	Allergic rhinitis, nasal septum deviation
60	M	Nasal obstruction	Otolaryngologist	Left septum deviation
86	M	Turbinate congestion, headache	Otolaryngologist	Headache
60	M	Rhinorrhea, nasal obstruction, night snores	Otolaryngologist	Chronic sinusitis

Age	Sex	Signs and symptoms	Specialty	Indication
33	F	Systemic lupus erythematosus with febrile neutropenia	General practitioner	Pneumonia
76	F	headache	Otolaryngologist	Osteoma (benign bone tumor) in the left frontal sinus. Chronic sinusitis.
68	F	Preoperative evaluation of the nasal region for 3-dimensional reconstruction	General surgeon	Nasal sequelae after skin malignant neoplasm surgery
66	F	Clinical picture compatible with Parotitis	Head and Neck Surgeon	Parotitis, thyroid papillary carcinoma treated with surgery and iodine therapy.
76	M	Asymptomatic	Head and Neck Surgeon	Previously operated squamous cell carcinoma of the face, evolving with salivary fistula
67	M	Pain, edema and drainage of purulent secretion in the upper gums, diabetes mellitus	General practitioner	Abscess in upper gum
60	F	Hyposmia, hypogeusia, hoarseness, headache	Neurologist	Hyposmia
77	M	Basal cell carcinoma of the inner corner of the right orbit	Head and Neck Surgeon	Basal cell carcinoma of the right orbit submitted to exenteration and complementary radiotherapy
79	F	Cough, fever, asthenia, hyporexia, dysphonia, dysphagia	General practitioner	Non-Hodgkin's lymphoma with oropharyngeal lesion
60	M	Ulcerated lesion on right lower eyelid	General practitioner	Basal cell carcinoma in the right lower eyelid
68	M	Fever of undetermined origin. Polyarthralgia, peripheral eosinophilia.	General practitioner	Fever of undetermined origin. Paraneoplasia? Vasculitis?
80	F	Right ear otalgia, pain when squeezing the skin over the temporomandibular joint	Otolaryngologist	Right ear otalgia, assessment of temporomandibular joint
92	M	Dysphagia, videolaryngoscoia showed an infiltrative lesion in the right piriform sinus with laryngeal wall edema, without airway obstruction	Geriatrician	Oropharyngeal malignant neoplasm

Table S4. Maxillofacial bone alterations in former Hansen's disease patients with full or partial rhinomaxillary syndrome (RMS)

Feature	Finding	No RMS (n=8)	Partial RMS (n=4)	Full RMS (n=4)	p-value (Full or partial RMS *vs.* no RMS)‡
**Osteitis**	No	8 (100.0%)	3 (75.0%)	3 (75.0%)	0.233
	Yes	0 (0.0%)	1 (25.0%)	1 (25.0%)	
**Nasal bones**	None	5 (62.5%)	1 (25.0%)	0 (0.0%)	0.217
	Mild-to-moderate	2 (25.0%)	1 (25.0%)	2 (50.0%)	
	Severe	1 (12.5%)	2 (50.0%)	2 (50.0%)	
**Anterior nasal spine**	None	3 (37.5%)	1 (25.0%)	0 (0.0%)	0.069
	Mild-to-moderate	4 (50.0%)	2 (50.0%)	0 (0.0%)	
	Severe	1 (12.5%)	1 (25.0%)	4 (100.0%)	
**Anterior nasal aperture**	None	6 (75.0%)	0 (0.0%)	0 (0.0%)	<0.001
	Mild-to-moderate	2 (25.0%)	3 (75.0%)	0 (0.0%)	
	Severe	0 (0.0%)	1 (25.0%)	4 (100.0%)	
**Nasal septum**	None	8 (100.0%)	4 (100.0%)	0 (0.0%)	0.001
	Mild-to-moderate	0 (0.0%)	0 (0.0%)	1 (25.0%)	
	Severe	0 (0.0%)	0 (0.0%)	3 (75.0%)	
**Inferior nasal turbinates**	None	5 (62.5%)	3 (75.0%)	0 (0.0%)	0.119
	Mild-to-moderate	3 (37.5%)	1 (25.0%)	3 (75.0%)	
	Severe	0 (0.0%)	0 (0.0%)	1 (25.0%)	
**Middle nasal turbinates**	None	7 (87.5%)	2 (50.0%)	0 (0.0%)	0.587
	Mild-to-moderate	1 (12.5%)	1 (25.0%)	3 (75.0%)	
	Severe	0 (0.0%)	1 (25.0%)	1 (25.0%)	
**Hard palate**	Normal	4 (50.0%)	2 (50.0%)	2 (50.0%)	1.00
	Abnormal	4 (50.0%)	2 (50.0%)	2 (50.0%)	
**Alveolar process of maxilla (anterior)**	None	0 (0.0%)	0 (0.0%)	0 (0.0%)	0.804
	Mild-to-moderate	3 (37.5%)	2 (50.0%)	3 (75.0%)	
	Severe	5 (62.5%)	2 (50.0%)	1 (25.0%)	
**Alveolar process of maxilla (posterior)**	None	0 (0.0%)	1 (25.0%)	1 (25.0%)	0.625
	Mild-to-moderate	4 (50.0%)	1 (25.0%)	2 (50.0%)	
	Severe	4 (50.0%)	2 (50.0%)	1 (25.0%)	
**Maxillofacial score**	median (IQR)	5.5 (4.5 - 7)	7.5 (6 - 10)	12 (11 - 14.5)	0.007

‡ Fisher's exact (categorical variables) or Kruskal-Wallis test (maxillofacial score)

Table S5. Rhinoscopy and nasal endoscopy findings in former Hansen's disease patients with full or partial rhinomaxillary syndrome (RMS)

Feature	Finding	No RMS (n=8)	Partial RMS (n=4)	Full RMS (n=4)	p-value (Full or partial RMS *vs.* no RMS)‡
**Nasal floor (integrity)**	Normal	8 (100.0%)	4 (100.0%)	4 (100.0%)	-
	Perforated	0 (0.0%)	0 (0.0%)	0 (0.0%)	
**Nasal floor (crusting)**	Not crusted	5 (62.5%)	4 (100.0%)	2 (50.0%)	0.487
	Crusted	3 (37.5%)	0 (0.0%)	2 (50.0%)	
**Nasal floor (secretion)**	None	7 (87.5%)	4 (100.0%)	4 (100.0%)	1.000
	Hyaline	0 (0.0%)	0 (0.0%)	0 (0.0%)	
	Mucous	1 (12.5%)	0 (0.0%)	0 (0.0%)	
	Purulent	0 (0.0%)	0 (0.0%)	0 (0.0%)	
**Nasal floor (polyps)**	None	8 (100.0%)	4 (100.0%)	4 (100.0%)	-
	Present	0 (0.0%)	0 (0.0%)	0 (0.0%)	
**Nasal septum**	Centred	5 (62.5%)	4 (100.0%)	0 (0.0%)	0.002
	Deviation 1-3	3 (37.5%)	0 (0.0%)	0 (0.0%)	
	Deviation 4-5	0 (0.0%)	0 (0.0%)	0 (0.0%)	
	Perforated 1-3	0 (0.0%)	0 (0.0%)	3 (75.0%)	
	Perforated 4-5	0 (0.0%)	0 (0.0%)	1 (25.0%)	
**Inferior nasal turbinates (trophism)**	Normotrophic	5 (62.5%)	2 (50.0%)	0 (0.0%)	0.178
	Hypertrophic	0 (0.0%)	0 (0.0%)	0 (0.0%)	
	Atrophic	3 (37.5%)	2 (50.0%)	4 (100.0%)	
**Inferior nasal turbinates (colour)**	Normal	2 (25.0%)	3 (75.0%)	2 (50.0%)	0.587
	Pale	5 (62.5%)	1 (25.0%)	2 (50.0%)	
	Hyperaemic	1 (12.5%)	0 (0.0%)	0 (0.0%)	
**Inferior nasal turbinates (crust or polyp)**	None	18 (85.7%)	12 (75.0%)	2 (50.0%)	0.508
	Crust	3 (14.3%)	4 (25.0%)	2 (50.0%)	
	Polyp	0 (0.0%)	0 (0.0%)	0 (0.0%)	
**Middle nasal turbinates (trophism)**	Normotrophic	5 (62.5%)	3 (75.0%)	0 (0.0%)	0.143
	Hypertrophic	0 (0.0%)	0 (0.0%)	0 (0.0%)	
	Atrophic	3 (37.5%)	1 (25.0%)	4 (100.0%)	
**Middle nasal turbinates (colour)**	Normal	2 (25.0%)	3 (75.0%)	2 (50.0%)	0.587
	Pale	5 (62.5%)	1 (25.0%)	2 (50.0%)	
	Hyperaemic	1 (12.5%)	0 (0.0%)	0 (0.0%)	
**Middle nasal turbinates (crust or polyp)**	None	8 (100.0%)	3 (75.0%)	3 (75.0%)	0.233
	Crust	0 (0.0%)	0 (0.0%)	1 (25.0%)	
	Polyp	0 (0.0%)	1 (25.0%)	0 (0.0%)	
**Inferior meatus**	Free	8 (100.0%)	3 (75.0%)	2 (50.0%)	0.100
	Crust	0 (0.0%)	1 (25.0%)	2 (50.0%)	
	Secretion	0 (0.0%)	0 (0.0%)	0 (0.0%)	
	Polyp	0 (0.0%)	0 (0.0%)	0 (0.0%)	
**Middle meatus**	Free	7 (87.5%)	3 (75.0%)	3 (75.0%)	0.500
	Crust	0 (0.0%)	0 (0.0%)	1 (25.0%)	
	Secretion	1 (12.5%)	0 (0.0%)	0 (0.0%)	
	Polyp	0 (0.0%)	1 (6.3%)	0 (0.0%)	
**Otorhinolaryngological score**	median (IQR)	3 (2 - 4.5)	1 (0 - 4.5)	5.5 (5 - 7)	0.142

‡ Fisher's exact (categorical variables) or Kruskal-Wallis test (otorhinolaryngological examination score)

Table S6. Oroscopic findings in former Hansen's disease patients with full or partial rhinomaxillary syndrome (RMS)

Feature	Finding	No RMS (n=8)	Partial RMS (n=4)	Full RMS (n=4)	p-value (Full or partial RMS *vs.* no RMS)
**Oral mucosa**	Normal	8 (100.0%)	4 (100.0%)	4 (100.0%)	-
	Pale	0 (0.0%)	0 (0.0%)	0 (0.0%)	
	Hyperaemic	0 (0.0%)	0 (0.0%)	0 (0.0%)	
**Palate**	Normal	7 (87.5%)	4 (100.0%)	4 (100.0%)	1.000
	Ogival	1 (12.5%)	0 (0.0%)	0 (0.0%)	
	Medium hard perforation	0 (0.0%)	0 (0.0%)	0 (0.0%)	
	Paramedium hard perforation	0 (0.0%)	0 (0.0%)	0 (0.0%)	
	Medium soft perforation	0 (0.0%)	0 (0.0%)	0 (0.0%)	
	Paramedium soft perforation	0 (0.0%)	0 (0.0%)	0 (0.0%)	
**Mouth (Hansen's disease nodules)**	None	8 (100.0%)	4 (100.0%)	4 (100.0%)	-
	Present	0 (0.0%)	0 (0.0%)	0 (0.0%)	
**Oropharynx (Hansen's disease nodules)**	None	8 (100.0%)	4 (100.0%)	4 (100.0%)	-
	Present	0 (0.0%)	0 (0.0%)	0 (0.0%)	
**Tongue**	Normotrophic	7 (87.5%)	4 (100.0%)	4 (100.0%)	1.000
	Atrophic	1 (12.5%)	0 (0.0%)	0 (0.0%)	
	Geographic	0 (0.0%)	0 (0.0%)	0 (0.0%)	
**Uvula**	Normal	8 (100.0%)	3 (75.0%)	4 (100.0%)	0.500
	Bifid	0 (0.0%)	0 (0.0%)	0 (0.0%)	
	Elongated	0 (0.0%)	1 (25.0%)	0 (0.0%)	
**Amygdala**	I/II	8 (100.0%)	4 (100.0%)	4 (100.0%)	-
	III/IV	0 (0.0%)	0 (0.0%)	0 (0.0%)	
	Tonsillectomy	0 (0.0%)	0 (0.0%)	0 (0.0%)	
**Tonsil pillar (colour)**	Normal	8 (100.0%)	3 (75.0%)	4 (100.0%)	0.500
	Pale	0 (0.0%)	0 (0.0%)	0 (0.0%)	
	Hyperaemic	0 (0.0%)	1 (25.0%)	0 (0.0%)	
**Dental disease**	None	2 (25.0%)	1 (25.0%)	3 (75.0%)	0.399
	Present	6 (75.0%)	3 (75.0%)	1 (25.0%)	
**Upper incisors**	All present	0 (0.0%)	0 (0.0%)	0 (0.0%)	0.771
	Loss of 1	0 (0.0%)	0 (0.0%)	0 (0.0%)	
	Loss of 2	0 (0.0%)	0 (0.0%)	0 (0.0%)	
	Loss of 3	2 (25.0%)	0 (0.0%)	1 (25.0%)	
	Loss of 4	6 (75.0%)	4 (100.0%)	3 (75.0%)	
**Number of upper decayed teeth†**	median (IQR)	0 (0 - 2)	0 (0 - 0)	0 (0 - 5)	0.766
**Number of missing upper teeth†**	median (IQR)	16 (14 - 16)	16 (16 - 16)	16 (13 - 16)	0.766
**Oroscopic examination score**	median (IQR)	2 (1.5 - 2)	2 (2 - 2.5)	1 (1 - 1.5)	0.142

‡ Fisher's exact (categorical variables) or Kruskal-Wallis test (number of decayed or missing upper teeth, oroscopic examination score)

† Not included in oroscopic examination score - all other features included, scored as 0 for none/normal, 1 for any other finding
